# Role of Periostin in the Development of Nasal Hyperresponsiveness in Mice with Allergic Rhinitis

**DOI:** 10.3390/ijms27031151

**Published:** 2026-01-23

**Authors:** Yukika Adachi, Yusuke Ando, Kanade Nagaosa, Moeka Maeno, Michio Yamashita, Fumiko Takenoya, Seiji Shioda, Motohiko Hanazaki, Hiroyasu Sakai, Yoshihiko Chiba

**Affiliations:** 1 Laboratory of Molecular Biology and Physiology, School of Pharmacy, Hoshi University, 2-4-41 Ebara, Shinagawa-ku, Tokyo 142-8501, Japan; ds2507@hoshi.ac.jp (Y.A.); s201187@hoshi.ac.jp (K.N.); s221224@hoshi.ac.jp (M.M.); 2 Laboratory of Clinical Pathology, Faculty of Pharmacy, Josai University, 1-1 Keyakidai, Sakado 350-0295, Saitama, Japan; y-ando@josai.ac.jp; 3 Laboratory of Sports Sciences, School of Pharmacy, Hoshi University, 2-4-41 Ebara, Shinagawa-ku, Tokyo 142-8501, Japan; yamashita.michio@hoshi.ac.jp (M.Y.); kuki@hoshi.ac.jp (F.T.); 4 Laboratory of Functional Morphology, Shonan University of Medical Sciences, 16–48 Kamishinano, Totsuka-ku, Yokohama 224-0806, Kanagawa, Japan; seiji.shioda@sums.ac.jp; 5 Department of Anesthesiology and Intensive Care Medicine, School of Medicine, International University of Health and Welfare, 4-3, Kozunomori, Narita 286-8686, Chiba, Japan; motohiko.hanazaki@gmail.com; 6 Laboratory of Toxicology, School of Pharmacy, Hoshi University, 2-4-41 Ebara, Shinagawa-ku, Tokyo 142-8501, Japan; sakai@hoshi.ac.jp

**Keywords:** allergic rhinitis, Japanese cedar pollen, nasal hyperresponsiveness, periostin

## Abstract

Periostin is a matricellular protein induced by type 2 cytokines. It has been shown to play important roles in airway inflammation and tissue remodeling. Although periostin has been studied in asthma and chronic rhinosinusitis, its role in allergic rhinitis (AR) and nasal hyperresponsiveness (NHR) is unclear. This study aimed to determine whether periostin is involved in the development of NHR in AR. A murine AR model was established by sensitization and repeated intranasal challenges with Japanese cedar pollen (JCP). In this animal model of AR, an increase in nasal responsiveness to histamine was observed 24 h after the last JCP challenge, indicating the development of NHR. RT-qPCR analysis revealed that the JCP-induced NHR was accompanied by increased periostin gene expression. Immunohistochemical examinations demonstrated the expression of integrin subunits α_V_ (Itgav), β_3_ (Itgb3) and β_5_ (Itgb5), which are known as receptors for periostin, in the nasal mucosa, especially in the mucosal epithelium. Notably, repeated intranasal administration of recombinant periostin to healthy I mice reproduced the NHR phenotype, as observed in AR model mice. These findings suggest that periostin upregulation in the nasal mucosa plays a causal role in the development of NHR, a key feature of AR.

## 1. Introduction

In recent decades, the prevalence of allergic rhinitis (AR) has been increasing worldwide [1], including in Japan. Sneezing, nasal itching, nasal congestion, and clear rhinorrhea are characteristic symptoms in people with AR. These symptoms result from complex immune responses, particularly those driven by type 2 helper T (Th2) cells, which secrete cytokines, such as interleukin-4 (IL-4) and IL-13. These cytokines mediate eosinophilic inflammation, mucus hypersecretion, and tissue remodeling in the nasal mucosa [2]. While AR itself is not fatal, the symptoms reduce quality of life and severely limit social activities. The pollinosis induced by Japanese cedar pollen (JCP) is the most common seasonal AR in Japan.

Periostin, encoded by the *Postn* gene, is a matricellular protein. It has been recognized as a critical mediator of Th2-driven inflammation and tissue remodeling. Periostin expression is induced by IL-4 and IL-13 in various cell types, including fibroblasts and epithelial cells [3,4,5]. This suggests that periostin is a potential link between Th2 cytokine signaling and structural changes in allergic tissues. In the lower airway, periostin has been implicated in promoting airway hyperresponsiveness, subepithelial fibrosis, and the recruitment of inflammatory cells. In a murine asthma model, lack of *Postn* resulted in reduced airway inflammation and hyperresponsiveness, highlighting its functional importance [6]. Our previous microarray analysis revealed significantly increased *Postn* expression in a murine model of allergic asthma (3.47 fold increase when compared to control mice, padj = 3.73 × 10^−8^) (GEO Accession No. GSE116504; probe ID: A_51_P489192 [7,8]). In asthmatic children, a high periostin level associated with poor asthma control has been reported [9].

While the role of periostin in asthma and chronic rhinosinusitis has been relatively well studied, its contribution to the pathogenesis of AR remains unclear. Clinical studies in humans suggest that periostin may serve as a biomarker for nasal inflammation. Several reports have found elevated periostin expression in nasal polyp tissues, as well as an association between tissue periostin and eosinophilic inflammation in chronic rhinosinusitis with nasal polyps [10,11,12]. Furthermore, serum periostin levels correlate with AR severity in some patient populations, supporting its relevance in upper airway allergic disease [13,14]. However, these clinical observations do not fully clarify whether periostin is causally involved in the pathogenesis of AR, nor do they address its regulation in nasal tissues under controlled experimental conditions.

The present study Investigated the”chan’e In *Postn* expression in the nasal mucosa of a murine AR model induced by Japanese cedar (*Cryptomeria japonica*) pollen (JCP), a major seasonal allergen in Japan. The effects of intranasal administration of recombinant periostin on nasal symptoms and histamine responsiveness in healthy I mice were also examined. In addition, the effects of recombinant periostin on the expression of multiple genes were also assessed in cultured human nasal epithelial cells (hNECs).

## 2. Results

### 2.1. Allergic Rrhinitis (AR) Symptoms and Nasal Hyperresponsiveness (NHR) Induced by JCP Challenge

**Figure 1:**
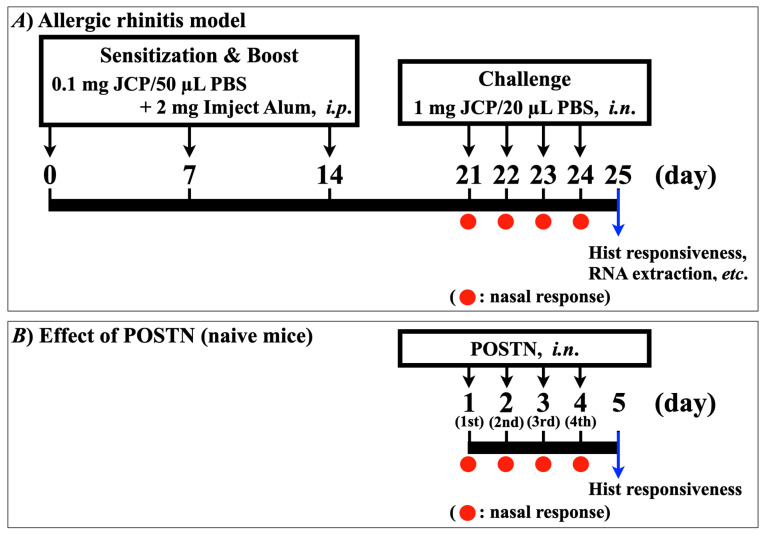
(**A**) **Schedules for immunization and repeated challenges with Japanese cedar pollen (JCP) in the mice.** Male ICR mice were sensitized by *i.p*. injections of JCP (0.1 mg in 50 µL PBS) with 2 mg Imuject Alum on days 0, 7, and 14. On days 21–24, animals were challenged intranasally (*i.n*.) with JCP (1 mg in 20 µL PBS, 10 µL/nostril) under the conscious state every 24 h. On day 25 (24 h after the last JCP challenge), the nasal responsiveness (sneezing/nasal rubbing) to intranasally administered histamine (Hist; 1–30 mM in saline, 10 µL/nostril) was measured under the conscious state. (**B**) **Schedules for *i.n*. treatments with recombinant mouse periostin (Postn) in I mice.** I male ICR mice were *i.n*. treated with Postn (10, 100, or 1000 ng/mL) or its vehicle PBS (5 µL/nostril, respectively) once daily for four consecutive days. Twenty-four hours after the last Postn treatment, the Hist responsiveness (sneezing/nasal rubbing) was measured as described above.

**Figure 2:**
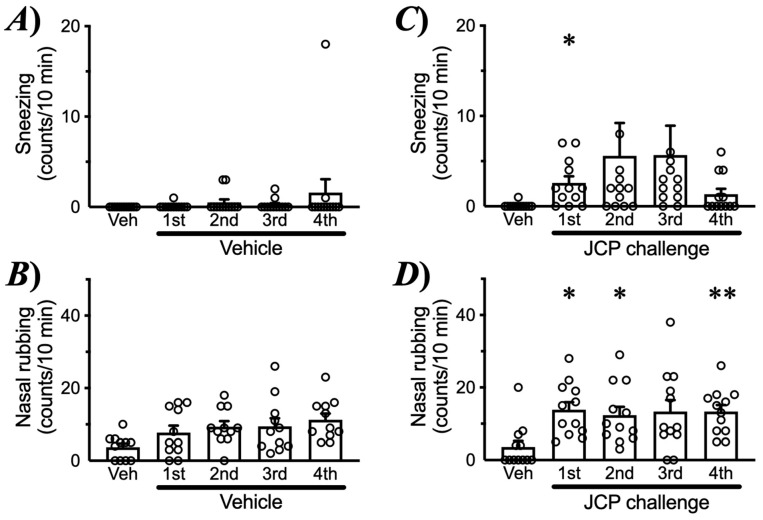
**Sneezing and nasal rubbing induced by Japanese cedar pollen (JCP) challenge.** Male ICR mice were sensitized by *i.p*. injections of JCP (0.1 mg in 50 µL PBS) with 2 mg Imuject Alum on days 0, 7, and 14. On days 21–24 (1st–4th, respectively), animals were intranasally challenged with JCP (1 mg in 20 µL PBS, 10 µL/nostril) or its vehicle (Veh) under the conscious state every 24 h. The sneezing (**A**,**C**) and nasal rubbing (**B**,**D**) induced by JCP (**C**,**D**) and its vehicle (**A**,**B**) were counted for 10 min. Results are presented as mean ± SEM from 12 animals. * *p* < 0.05 and ** *p* < 0.01 vs. Veh by one-way ANOVA with post hoc Dunnett’s multiple comparisons test.

**Figure 3:**
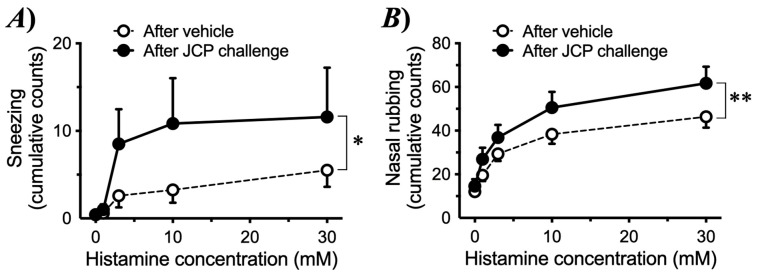
**Nasal hyperresponsiveness to histamine induced by Japanese cedar pollen (JCP) challenge.** Male ICR mice were sensitized by *i.p*. injections of JCP (0.1 mg in 50 µL PBS) with 2 mg Imuject Alum on days 0, 7, and 14. On days 21–24, animals were intranasally challenged with JCP (1 mg in 20 µL PBS, 10 µL/nostril) under the conscious state every 24 h. Twenty-four hours after the last JCP challenge (day 25), sneezing (**A**) and nasal rubbing (**B**) induced by intranasally administered histamine (1–30 mM in saline, 10 µL/nostril) were counted for 10 min. Results are presented as mean ± SEM from 12 animals. * *p* < 0.05 and ** *p* < 0.01 by two-way ANOVA with post hoc Tukey’s multiple comparisons test.

**Figure 4:**
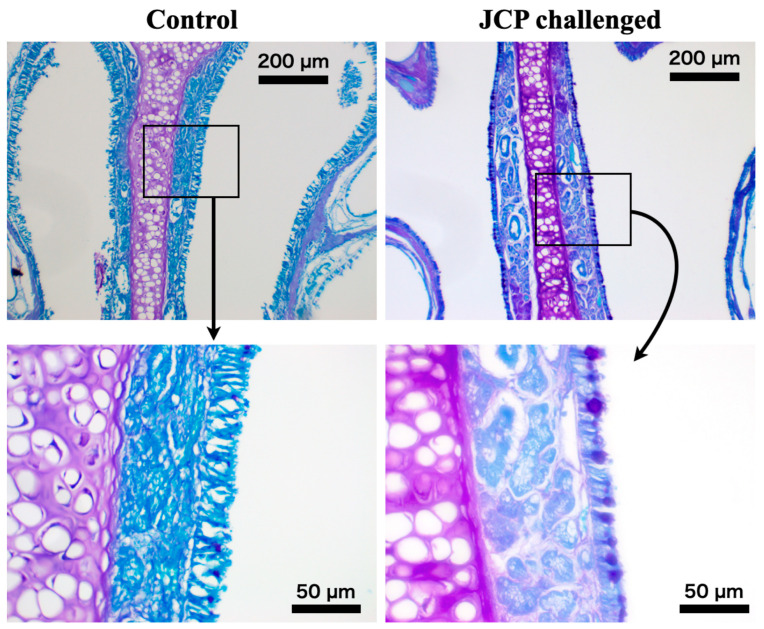
**Histological change in nasal mucosa induced by repeatedly challenged with Japanese cedar pollen (JCP) in the mouse.** Male ICR mice were sensitized by *i.p*. injections of JCP (0.1 mg in 50 µL PBS) with 2 mg Imuject Alum on days 0, 7, and 14. On days 21–24, animals were intranasally challenged with JCP (1 mg in 20 µL PBS, 10 µL/nostril) under the conscious state every 24 h. Twenty-four hours after the last JCP challenge (day 25), the nasal tissues were isolated and subjected to histological examinations with periodic acid–Schiff (PAS) staining in paraffin-embedded sections (4 µm thickness). Cont: sensitized control, and Chal: repeatedly JCP-challenged animals. Scale bars: 200 µm. The photos are representative of three independent experiments, respectively.

**Figure 5:**
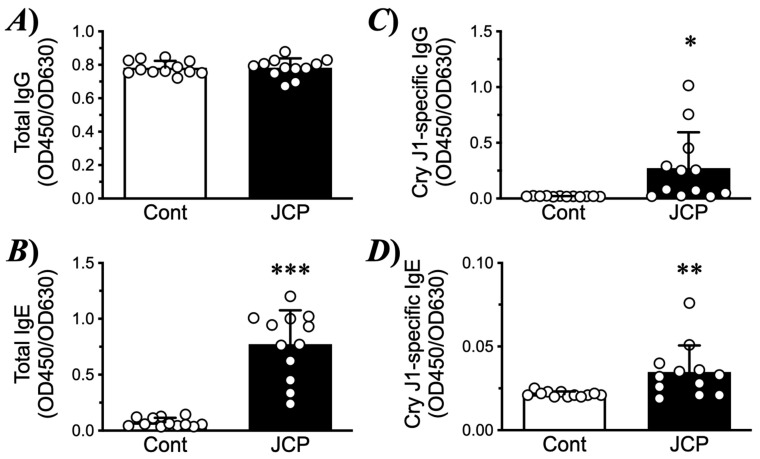
**Changes in the levels of total immunoglobulin G (total IgG: (A)), IgE (total IgE: (B)), Cry J1-specific IgG (C), and Cry J1-specific IgE (D) in sera of Japanese cedar pollen (JCP) challenged mice.** The sera obtained from the control (Cont) and the JCP-challenged mice were assayed for total and Cry J1-specific IgG and IgE by ELISA. Results are presented as mean ± SEM from 12 animals. * *p* < 0.05, ** *p* < 0.01 and *** *p* < 0.001 by unpaired Student’s *t*-test.

To induce nasal allergic response, mice were sensitized by intraperitoneal injections of JCP plus alum on days 0, 7, and 14, and subsequently challenged by intranasal (*i.n*.) administrations of JCP on days 21–24 (Figure 1A). Each nasal JCP challenge resulted in an increase in the frequencies of sneezing and nasal rubbing (Figure 2), indicating that nasal allergic response had been induced. In this mouse model of AR, the frequencies of sneezing and nasal rubbing elicited by cumulative *i.n*. administrations of histamine (1–30 mM, 10 µL/nostril; each for 10 min) were significantly increased by day 25 (24 h after the last JCP challenge) compared with those in vehicle-treated animals (Figure 3), indicating that NHR had been induced after repeated JCP challenges. Histochemical examinations also revealed increased PAS-positive staining in the nasal epithelial cells of mice repeatedly challenged with JCP (Figure 4). Increases in Cry J1-specific IgE and IgG in sera were also observed in the AR mice (Figure 5). These findings suggest that nasal JCP challenges to sensitized mice caused an allergic reaction in the nasal mucosa, resulting in nasal inflammation and NHR.

### 2.2. Changes in Gene Expression of Periostin and Integrin Subunits in the Nasal Mucosal Tissues of Mice Repeatedly Challenged with JCP

**Figure 6:**
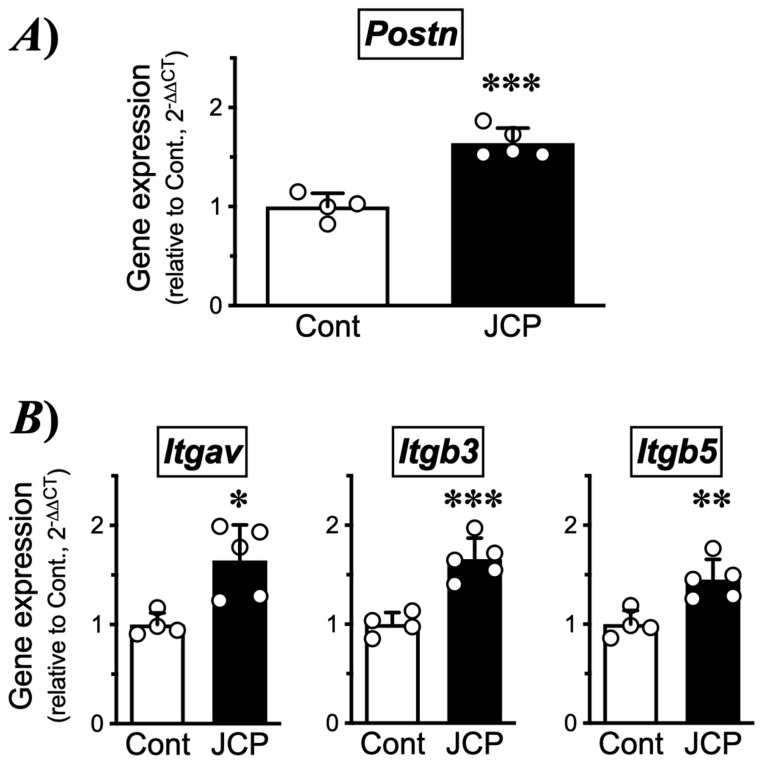
**Changes in the gene expression of periostin (*Postn*: (A)) and its receptor subunits (B) in nasal mucosae of the Japanese cedar pollen (JCP)-challenged mice.** Total RNA sample of each mouse (4 animals/sample, respectively) was subjected to RT-qPCR analysis and relative expression of respective target genes to *Gapdh* were calculated by the 2^−ΔΔCT^ methods. Results are presented as mean ± SEM from 4 (Cont) and 5 (JCP) independent experiments. * *p* < 0.05, ** *p* < 0.01, and *** *p* < 0.001 by unpaired Student’s *t*-test.

**Figure 7:**
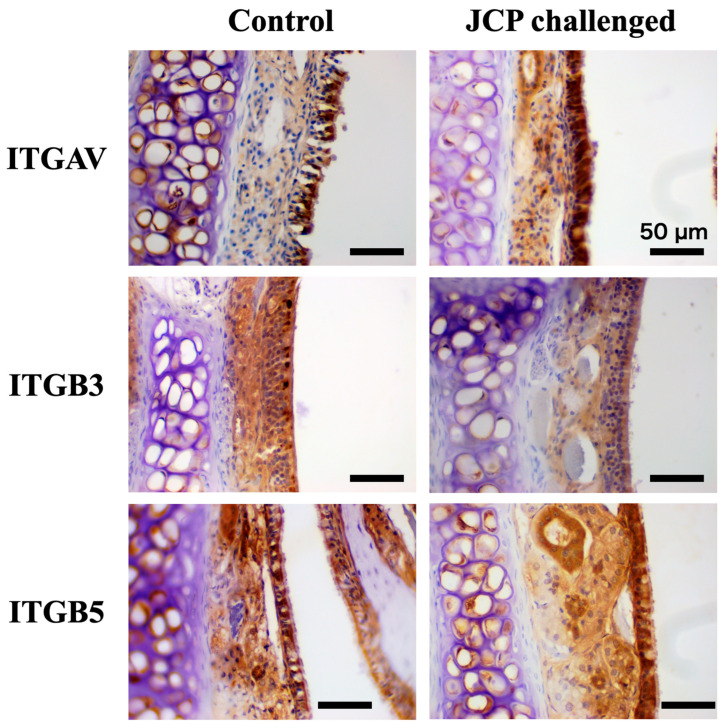
**Immunohistochemistry for periostin receptor subunits in nasal mucosae of control (left panels) and Japanese cedar pollen (JCP)-challenged mice (right panels).** Male ICR mice were sensitized by *i.p*. injections of JCP (0.1 mg in 50 µL PBS) with 2 mg Imuject Alum on days 0, 7, and 14. On days 21–24, animals were intranasally challenged with JCP (1 mg in 20 µL PBS, 10 µL/nostril) or its vehicle (PBS: control) under the conscious state every 24 h. Twenty-four hours after the last JCP challenge (day 25), the nasal tissues were isolated and subjected to immunohistochemical examinations with anti-integrin alpha v (Itgav: 1:300 dilution; **upper panels**), anti-integrin beta 3 (Itgb3: 1:300 dilution; **middle panels**), and anti-integrin beta 5 (Itgb5: 1:400 dilution; **lower panels**) antibodies in paraffin-embedded sections (4 µm thickness). Scale bars: 50 µm.

Increasing evidence suggests that periostin expression is increased in allergic inflammation, including upper airways [10,11,12]. Periostin is known to be capable of binding integrins, such as α_V_β_3_ and α_V_β_5_ [15]. So next, changes in the gene expression of periostin (*Postn*) and integrin subunits (*Itgav*, *Itgb3* and *Itgb5*) in nasal mucosae of the AR mice with NHR were determined. As shown in (Figure 6A), the RT-qPCR analysis revealed a significant increase in *Postn* in the nasal mucosa 24 h after the last JCP challenge. In addition, significant increases in genes for integrin subunits, *Itgav*, *Itgb3*, and *Itgb5*, were also found in the AR mice (Figure 6B). Immunohistochemical examinations demonstrated the expression of integrin subunits α_V_ (Itgav), β_3_ (Itgb3), and β_5_ (Itgb5) in the nasal mucosa, especially in the mucosal epithelium (Figure 7). These findings indicate that periostin can act directly on nasal epithelial cells and that the periostin-mediated signaling is augmented in the AR mice.

### 2.3. Effects of i.n. Treatments with Periostin on Nasal Response in Healthy Naive Mice

**Figure 8:**
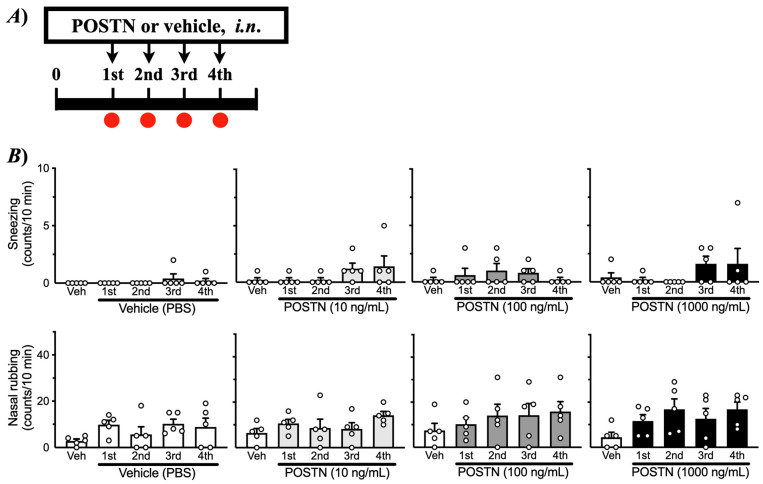
**Nasal response induced by periostin (Postn) in naive mice.** Male ICR mice were challenged by intranasal (*i.n*.) administrations of recombinant mouse Postn (0–1000 ng/mL in PBS, 5 µL/nostril) every 24 h for 4 consecutive days (1st–4th) under the conscious state (**A**). Each day, the sneezing (**upper panels**) and nasal rubbing (**lower panels**) were counted for 10 min immediately after the *i.n*. administration (**B**). Results are presented as mean ± SEM from 5 animals.

**Figure 9:**
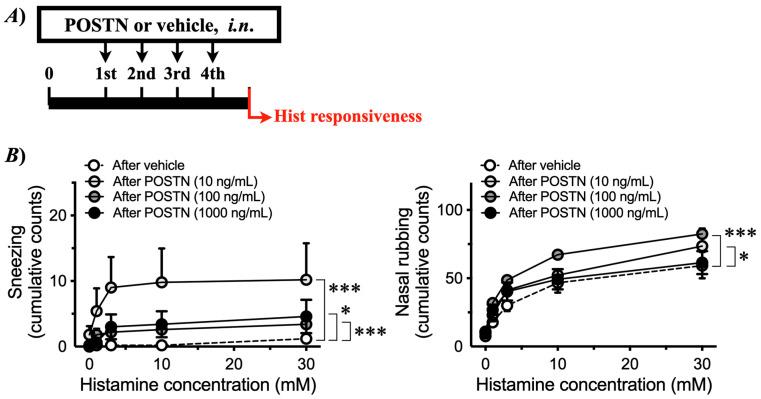
**Effects of periostin (Postn) on nasal responsiveness to histamine in naive mice.** Male ICR mice were challenged by intranasal (*i.n*.) administrations of recombinant mouse Postn (0–1000 ng/mL, 5 µL/nostril, respectively) every 24 h for 4 consecutive days under the conscious state (**A**). Twenty-four hours after the last Postn administration, sneezing (**left panel**) and nasal rubbing (**right panel**) induced by intranasally administered Hist (0–30 mM in saline, 10 µL/nostril) were counted for 10 min (**B**). Results are presented as mean ± SEM from 5 animals. * *p* < 0.05 and *** *p* < 0.001 vs. after vehicle by two-way ANOVA with Tukey multiple comparisons test.

To determine the causal role of periostin in NHR, healthy naive mice were treated with recombinant mouse periostin intranasally once daily for four consecutive days (Figure 1B). Periostin is a matricellular protein that primarily acts as a dynamic modulator of cell–matrix signaling rather than as a static structural component. The present periostin administration experiment was designed to assess functional sufficiency rather than to precisely mimic endogenous matrix deposition. As shown in Figure 8, periostin itself did not elicit any symptomatic response, such as an increase in the frequency of sneezing or nasal rubbing. However, the repeated *i.n*. administration of periostin caused a significant increase in histamine-induced sneezing or nasal rubbing (Figure 9), indicating that periostin can induce the NHR, as observed in AR model mice (Figure 3).

### 2.4. Effects of Periostin on Multiple Gene Expression in Human Nasal Epithelial Cells (hNECs)

**Figure 10:**
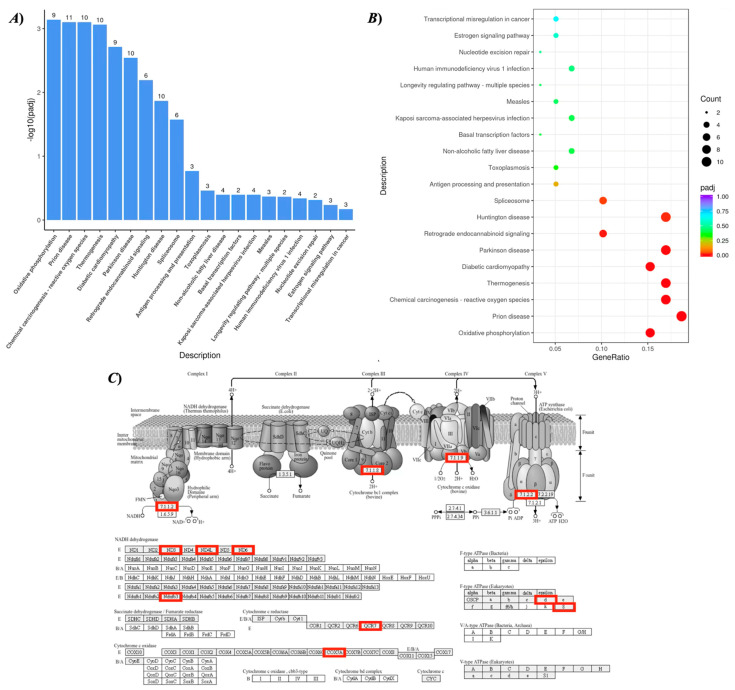
**Kyoto Encyclopedia of Genes and Genomes (KEGG) pathway analysis of upregulated genes in periostin (Postn)-treated human nasal epithelial cells (hNECs) compared with control cells.** Cultured hNECs were treated with recombinant human Postn (100 ng/mL) or its vehicle PBS for 24 h. Total RNA sample was subjected to RNA-seq analysis, and KEGG pathway enrichment analysis of the differentially expressed genes was performed. (**A**) KEGG enrichment analysis histogram ranked by −log10(adjusted *p*-value: padj). The numbers on the bars indicate the gene counts associated with each pathway. (**B**) KEGG enrichment analysis scatter plot. The *x*-axis represents the gene ratio, and the color indicates the adjusted *p*-value (padj). The size of each circle corresponds to the number of genes associated with each pathway. (**C**). Schematic representation of the oxidative phosphorylation pathway (KEGG ID: hsa00190) showing upregulated genes in Postn-treated hNECs. Red boxes indicate the genes that were significantly upregulated in Postn-treated cells.

The RNA-seq analysis of hNECs stimulated with recombinant human periostin revealed that 190 genes in total were significantly changed (DESeq2 *p*-value ≤ 0.05, |log2FoldChange| ≥ 0.0): 94 genes were upregulated and 96 genes were downregulated. Because metabolic pathways typically involve coordinated yet modest changes in multiple genes [16,17], differential expression was evaluated using statistical significance together with directionality. Pathway enrichment analyses were performed based on this gene set. Periostin caused significant upregulation of several genes encoding components of the mitochondrial respiratory chain, including *MT-ND4L*, *ATP5PD*, *MT-ND6*, *MT-ATP8*, *UQCRB*, *NDUFB3*, *MT-ND3*, *COX7A2*, and *NDUFS4* (Table 1), accompanied by enrichment of the KEGG oxidative phosphorylation pathway (hsa00190) as the top enrichment pathway (Figure 10). These observations suggest that periostin could enhance mitochondrial metabolic activity in NECs.

## 3. Discussion

The current study demonstrated that JCP-sensitized mice exhibited pronounced sneezing and nasal rubbing following nasal JCP challenge (Figure 2). In addition, the enhanced responsiveness to histamine 24 h after the last JCP exposure (Figure 3) indicated the development of nasal hyperresponsiveness (NHR). In nasal mucosa of this animal model of AR with NHR, a significant increase in *Postn* expression was observed (Figure 6A). Notably, repeated intranasal administration of recombinant periostin to healthy naive mice reproduced the NHR phenotype (Figure 9), even though periostin alone did not induce sneezing or nasal rubbing (Figure 8). These findings strongly suggest that periostin upregulation in the nasal mucosa plays a causal role in the induction of NHR, a key feature of AR.

Allergic diseases are characterized not only by Th2-driven inflammation, but also by an exaggerated sensitivity in the target tissues, which plays a crucial role in symptom exacerbation. Airway hyperresponsiveness, an exaggerated bronchoconstrictive response to various non-specific stimuli, is a universal and defining feature of asthma [18]. Patients with allergic dermatitis exhibit exaggerated itch responses to pruritogenic stimuli and even to normally innocuous mechanical stimuli [19,20]. Similarly, patients with AR frequently exhibit NHR, a pathophysiological feature that exacerbates AR symptoms [21]. In the present study, the AR model sensitized and repeatedly challenged with JCP developed pronounced NHR, as demonstrated by significantly augmented responsiveness to histamine (Figure 3). This animal model may therefore be useful for elucidating the mechanisms underlying the development of NHR in AR.

The current study revealed an upregulation of *Postn* in the nasal mucosa 24 h after the last JCP challenge (Figure 6A), at which NHR was observed (Figure 3). This finding is consistent with previous reports showing increased periostin expression in allergic tissues, including nasal polyps, chronic rhinosinusitis, and asthmatic airways [22,23,24]. Periostin is a matricellular protein strongly induced by Th2 cytokines, such as IL-4 and IL-13 [5,25,26], and is well recognized as a key mediator in allergic airway inflammation and tissue remodeling [27]. A notable finding of the current study is that repeated intranasal administration of recombinant periostin to healthy naive mice reproduced the NHR (Figure 9). In contrast, periostin alone did not induce sneezing or nasal rubbing (Figure 8). The findings suggest that periostin itself is not a direct pruritogenic or irritant stimulus but rather functions as a “sensitizer” of the nasal mucosa, thereby augmenting nasal reactivity. Similar modulatory roles of periostin have been reported in models of allergic diseases, such as atopic dermatitis and bronchial asthma [6,24,28]. Thus, it is possible that periostin plays a key role in regulating tissue sensitivity in allergic diseases, including nasal mucosal tissue in AR.

The RNA-seq analysis of human nasal epithelial cells (hNECs) stimulated with recombinant periostin revealed significant upregulation in several genes that encode components of the mitochondrial respiratory chain (Table 1), accompanied by enrichment of the KEGG oxidative phosphorylation pathway (hsa00190: Figure 10). The results indicated that periostin might enhance mitochondrial metabolic activity in nasal epithelial cells. Because heightened oxidative phosphorylation pathway activity increases electron flux through the electron transport chain, it could lead to greater mitochondrial electron leakage and subsequent reactive oxygen species (ROS) production [29,30,31]. An increase in ROS has been associated with altered epithelial barrier properties, sensory signaling, and inflammatory responses [32,33]. ROS are increasingly recognized as signaling molecules capable of sensitizing transient receptor potential (TRP) channels, including TRPA1 and TRPV1 [34]. These channels are expressed in airway and nasal sensory neurons and have been implicated in airway hyperresponsiveness and neurogenic inflammation [35]. In parallel, heightened oxidative phosphorylation pathway activity can increase ATP availability. Extracellular ATP released from epithelial cells could activate purinergic receptors and augment neurogenic inflammation and sensory nerve excitability [36,37,38]. Since expression of these TRP channels TRPA1/TRPV1 [39,40] and purinergic receptors P2X/P2Y [41,42,43] has also been demonstrated in nasal epithelial cells, increased production of mitochondrial ROS/ATP may contribute to the development of NHR induced by periostin. Additionally, periostin interacts with integrins (e.g., α_V_β_3_, α_V_β_5_), which are also expressed in the nasal mucosa (Figure 7), to activate the PI3K/AKT pathway [44]. Activation of PI3K/AKT pathway is known to interact with mitochondrial biogenesis and metabolic activation [45]. Further studies are required to clarify the essential role of periostin in the development of NHR.

Periostin has been studied primarily as a biomarker of type 2 inflammation [46,47]. Preclinical studies suggest that blocking periostin or preventing its interaction with integrin receptors could ameliorate allergic inflammation [24,48], supporting the feasibility of periostin-targeted therapy in Th2-driven diseases. Neutralizing anti-periostin antibodies have been shown to improve airway inflammation and hyperresponsiveness in murine experimental asthma [6]. In a mouse model of atopic dermatitis, pharmacological inhibition of periostin-integrin interactions has been demonstrated to attenuate allergic skin inflammation [48]. While direct periostin-specific biologics for allergic diseases are not yet clinically available, periostin has been used to categorize responses to Th2-targeted therapies such as IL-13 blockade, where higher serum periostin levels correlated with greater treatment response [49]. It has also been suggested that serum periostin levels are correlated with the severity of AR in certain patient groups [13,14]. Collectively with the current observations, these findings suggest that strategies targeting periostin could be explored as future therapeutics for refractory AR with NHR, although direct clinical evidence linking serum or nasal periostin levels to the severity of NHR in AR patients remains limited.

In conclusion, the current study demonstrated that an increase in periostin in nasal mucosa is one of the causes of NHR in AR induced by JCP. Understanding the underlying mechanism of periostin-mediated changes in nasal mucosal signaling could lead to new approaches for treating AR.

### Limitations of the Study

While the present study provided evidence that periostin is sufficient to induce NHR, the downstream mechanisms linking periostin signaling to NHR remain to be fully elucidated. Our RNA-seq analysis revealed an enrichment of the oxidative phosphorylation pathway in periostin-stimulated hNECs, raising the possibility that periostin enhances mitochondrial activity. However, this interpretation remains speculative, as direct measurements of ROS production, ATP release, and/or receptor activation were not performed in the present study. Similarly, although the current study suggested increased expression and epithelial localization of the periostin-binding integrins α_v_β_3_ and α_v_β_5_ in the nasal mucosa, the necessity of integrin-mediated signaling for periostin-induced NHR has not yet been established. This should be addressed in future studies using pharmacological or genetic approaches. In addition, this study was conducted exclusively in male mice to minimize variability associated with hormonal cycles. Potential sex-dependent differences in JCP- and periostin-mediated nasal responses warrant further investigation. Another limitation of the present study is that additional control groups, such as non-sensitized mice challenged intranasally with JCP or non-sensitized mice challenged with vehicle for JCP, were not included. Inclusion of these control groups would provide further insight into the development of NHR, and should be addressed in future studies. From a translational perspective, our findings suggest that periostin could be a potential therapeutic target for severe, refractory AR. However, direct clinical evidence linking periostin to functional indices of NHR in patients remains limited. These considerations highlight the mechanistic implications of our findings and their current limitations, and emphasize the need for future studies to validate periostin-mediated pathways in NHR.

## 4. Materials and Methods

### 4.1. Animals and Treatments

Male ICR mice (weighing 33–36 g, 7-week-old upon arrival) purchased from Tokyo Laboratory Animals Science Co., Ltd. (Tokyo, Japan) were used. The mice were housed in a pathogen-free, temperature- and humidity-controlled facility on a 12/12 h light/dark cycle with ad libitum access to food and water. All animal experiments were approved by the Animal Care Committee of the Hoshi University (Approval No. P24-023: Tokyo, Japan). Animal studies are reported in compliance with the ARRIVE guidelines [50,51]. The specific sample size used in each individual study can be found in the respective graphs, figure legends or tables. To avoid hormonal cycle variability, male animals were used in the present study.

Preparation of a murine model of allergic rhinitis was performed by the method of Fukuoka et al. [52] with minor modifications. In brief, mice were actively sensitized by intraperitoneal (*i.p*.) injections of 200 µL of Japanese cedar pollen (JCP: Biostir Inc., Osaka, Japan) solution (2 mg/mL in PBS) mixed with 200 µL of Imject Alum (Pierce Biotechnology, Inc., Rockford, IL, USA) on days 0, 7, and 14. From day 21, the sensitized mice were challenged by intranasal (*i.n*.) administrations of JCP (50 mg/mL in PBS, 10 µL/nostril) for four consecutive days (Figure 1A).

In another series of experiments, healthy naive mice were treated with recombinant mouse periostin (ab276845, Abcam plc., Cambridge, UK) intranasally (0, 10, 100, or 1000 ng/mL; 5 µL/nostril, respectively) once daily for four consecutive days (Figure 1B). Twenty-four hours after the last periostin treatment, the histamine responsiveness was measured as described below.

### 4.2. Evaluation of Nasal Allergic Response Induced by JCP Challenge

Before each nasal JCP challenge, the mice were placed into a plastic cage (30 × 20 × 13 cm) for 10 min for acclimatization. Immediately after the *i.n*. instillation of JCP into the bilateral nasal cavities, the animals were returned to the plastic cage, and the frequencies of sneezing (a head movement accompanied by a contraction of the abdominal muscles) and nasal rubbing were counted for 10 min. Video recordings were also made during the observation periods, and behavioral changes were checked by another observer.

### 4.3. Evaluation of Nasal Responsiveness to Histamine and Sample Collection

Twenty-four hours after the last JCP challenge (day 25 in Figure 1A), the frequencies of sneezing and nasal rubbing induced by *i.n*. instillation of histamine (Hist; 1–30 mM in saline, 10 µL/nostril: FUJIFILM Wako Pure Chemical Corporation, Osaka, Japan) were counted cumulatively (each for 10 min) as described above. After the evaluation of histamine responsiveness, the mice were euthanized by overdose inhalation of isoflurane, and blood was collected from the heart. Serum was obtained by centrifugation at 800× *g* for 10 min at 4 °C and stored at −20 °C until use. Immediately after bleeding, the nasal mucosae of the septum and turbinates were isolated from the mice under a stereomicroscope for RNA extraction. Due to the limited amount of tissue obtained from a single mouse, mucosal tissues from four mice were pooled and lysed in 1 mL of TRIzol (Thermo Fisher Scientific, Waltham, MA, USA) to prepare one total RNA sample.

### 4.4. Measurements of Total and Cry J1-Specific IgG and IgE Antibodies

Total IgG levels in sera were measured by ELISA using goat anti-mouse IgG (BioLegend Inc., San Diego, CA, USA) as a capture antibody and horseradish peroxidase (HRP)-conjugated anti-mouse IgG (LGC Clinical Diagnostics, Inc., Milford, MA, USA) as a detection antibody. Total IgE levels in sera were measured using a mouse IgE ELISA kit (BioLegend Inc.) according to the manufacturer’s instructions. Serum levels of IgG and IgE specific for Cry J1, a major allergen of JCP, were also measured using 96-well plates that were pre-coated with purified Cry J1 protein (0.2 µg/mL, 100 µL/well at 4 °C overnight; BioDynamics Laboratory Inc., Tokyo, Japan).

### 4.5. Histochemical and Immunohistochemical Evaluation of Nasal Tissues

In another series of experiments, the nasal tissues of control and JCP-challenged mice were isolated and fixed in 4% (wt/vol) paraformaldehyde, and decalcified in K-CX solution (Falma Co. Ltd., Tokyo, Japan) for 4–5 days at 4 °C. The specimens were embedded in paraffin and sectioned at 4 µm thickness. Sections were deparaffinized and stained with periodic acid–Schiff (PAS) reagent (Vector Laboratories, Inc., Newark, CA, USA), and counterstained with Light Green Solution (Vector Laboratories, Inc.). The deparaffinized sections were also used for immunohistochemistry using IMMPRESS Horse Anti-Rabbit IgG Plus Polymer Kit (Vector Laboratories, Inc.) according to manufacturer’s instructions as previously described in detail [53,54]. The primary antibodies used were rabbit anti-integrin alpha v polyclonal antibody (27096-1-AP, 1:300 dilution; Proteintech Group, Inc., Rosemont, IL, USA), rabbit anti-integrin beta 3 polyclonal antibody (18309-1-AP, 1:300 dilution; Proteintech Group, Inc.), and rabbit anti-integrin beta 5 polyclonal antibody (28543-1-AP, 1:400 dilution; Proteintech Group, Inc.).

### 4.6. Cell Culture and Treatments

Normal human nasal epithelial cells (hNECs: C-12620; PromoCell GmbH, Heidelberg, Germany) were maintained in airway epithelial cell growth medium (C-21060; PromoCell) supplemented with 0.004 mL/mL bovine pituitary extract, 10 ng/mL human L-epidermal growth factor (hEGF), 5 µg/mL human insulin, 0.5 µg/mL hydrocortisone, 0.5 µg/mL adrenalin, 6.7 ng/mL triiodo-L-thyronine, 10 µg/mL human transferrin, 0.1 ng/mL retinoid acid, and Penicillin-Streptomycin-Amphotericin B Suspension (1 U/mL penicillin, 1 µg/mL streptomycin, 0.25 µg/mL amphotericin B: FUJIFILM Wako Pure Chemical Corporation, Osaka, Japan). Cells were maintained at 37 °C in a humidified atmosphere (5% CO_2_), fed every 48 to 72 h, and passaged when cells reached 80% confluence. Then the hNECs (passage 1) were seeded in 6-well plates (Thermo Fisher Scientific) at a density of 1 × 10^5^ cells/well. When ~95% confluence was observed, cells were treated with 100 ng/mL recombinant human periostin (ab310788; Abcam, Inc., Waltham, MA, USA) or their vehicle PBS. Twenty-four hours after the treatment, cells were immediately lysed in TRIzol (1 mL/well: Thermo Fisher Scientific) for RNA extraction.

### 4.7. Quantitative RT-PCR (RT-qPCR) Analyses

The gene expression levels were examined by real-time RT-PCR. In brief, cDNA was synthetized from total RNA using the ReverTra Ace^®^ qPCR RT Master Mix with gDNA Remover (Toyobo Co., Ltd., Tokyo, Japan) according to the manufacturer’s instructions. The resultant cDNA was then subjected to real-time PCR analysis using the THUNDERBIRD^®^ Next SYBR^™^ qPCR Mix (Toyobo Co., Ltd.) and a LightCycler^®^ 96 System (Roche Diagnostics, Tokyo, Japan). The gene-specific PCR primers were designed using the BLAST database. The primer sets used are listed in Table 2. Data were presented as expressions relative to *Gapdh* mRNA as a housekeeping gene using the 2^−ΔΔCT^ method as described previously [55,56,57,58,59].

### 4.8. RNA-Seq Analysis

Total RNA was extracted from human nasal epithelial cells using the PureLink RNA Mini Kit (Thermo Fisher Scientific) according to the manufacturer’s instructions. The RNA quality was assessed with an Agilent 2100 Bioanalyzer (Agilent Technologies, Santa Clara, CA, USA). Library preparation, sequencing, and primary data processing were performed by Novogene Co., Ltd. (Beijing, China). Raw sequencing reads were quality-controlled and aligned to the human reference genome (GRCh38). Gene-level read counts were generated, and transcript abundance was normalized and expressed as transcripts per million (TPM).

### 4.9. Data Availability Statement

The RNA-seq data generated in this study have been deposited in the NCBI Gene Expression Omnibus (GEO) under accession number GSE313641. Raw sequencing data are available through the NCBI Sequence Read Archive (SRA) and are linked to the GEO record.

### 4.10. Data and Statistical Analyses

All the data were expressed as the mean ± SEM. Statistical analyses were performed using GraphPad Prism software (Prism 10 for macOS ver. 10.5.0, GraphPad Software, San Diego, CA, USA). Prior to applying parametric statistical tests, the normality of data distribution was assessed using Shapiro–Wilk test. Statistical significance of difference was determined by unpaired Student’s *t*-test, one-way analysis of variance (ANOVA) with post hoc Tukey’s multiple comparisons test, or two-way ANOVA with post hoc Tukey’s multiple comparisons test, as appropriate. A value of *p* < 0.05 was considered statistically significant.

## Figures and Tables

**Table 1:** Upregulated genes involved in OXIDATIVE PHOSPHORYLATION pathway (KEGG ID: hsa00190) in periostin (Postn)-treated human nasal epithelial cells (hNECs) determined by RNA-seq analysis.

Gene Symbol	Gene ID	TPM (Mean ± SEM)		log2FC	*p* Value
		Cont	Postn		
*MT-ND4L*	ENSG00000212907	14,667 ± 256.30	16,214 ± 361.41	0.145	0.000
*ATP5PD*	ENSG00000167863	1308 ± 21.40	1498 ± 45.25	0.196	0.002
*MT-ND6*	ENSG00000198695	6983 ± 465.69	9127 ± 791.03	0.386	0.014
*MT-ATP8*	ENSG00000228253	395 ± 62.00	636 ± 78.70	0.690	0.016
*UQCRB*	ENSG00000156467	1606 ± 42.04	1790 ± 85.25	0.157	0.017
*NDUFB3*	ENSG00000119013	676 ± 16.01	769 ± 25.75	0.187	0.018
*MT-ND3*	ENSG00000198840	7595 ± 464.16	8334 ± 121.58	0.134	0.023
*COX7A2*	ENSG00000112695	1633 ± 33.42	1770 ± 32.21	0.116	0.042
*NDUFS4*	ENSG00000164258	588 ± 13.85	659 ± 16.99	0.164	0.048

Cultured hNECs were treated with recombinant human Postn (100 ng/mL) or its vehicle (PBS: Cont) for 24 h. Total RNA sample was subjected to RNA-seq analysis, and KEGG pathway enrichment analysis of the differentially expressed genes was performed. TPM: transcripts per million, SEM: standard error of the mean, FC: fold change, *MT-ND4L*: mitochondrially encoded NADH 4L dehydrogenase, *ATP5PD*: ATP synthase peripheral stalk subunit D, *MT-ND6*: mitochondrially encoded NADH dehydrogenase 6, *MT-ATP8*: mitochondrially encoded ATP synthase 8, *UQCRB*: ubiquinol-cytochrome c reductase binding protein, *NDUFB3*: NADH: ubiquinone oxidoreductase subunit B3, *MT-ND3*: mitochondrially encoded NADH dehydrogenase 3, *COX7A2*: cytochrome c oxidase subunit 7A2, and *NDUFS4*: NADH: ubiquinone oxidoreductase subunit S4.

**Table 2:** Primer sequences for RT-PCR used in the present study.

Gene Name	RefSeq Accession		Sequence	Amplicon Size
*Postn*	NM_015784	Sense	5′-CAGCAAACCACTTTCACCGACC-3′	
		Antisense	5′-AGAAGGCGTTGGTCCATGCTCA-3′	137 bp
*Itgav*	NM_008402	Sense	5′-GTGTGAGGAACTGGTCGCCTAT-3′	
		Antisense	5′-CCGTTCTCTGGTCCAACCGATA-3′	101 bp
*Itgb3*	NM_016780	Sense	5′-GTGAGTGCGATGACTTCTCCTG-3′	
		Antisense	5′-CAGGTGTCAGTGCGTGTAGTAC-3′	139 bp
*Itgb5*	NM_010580	Sense	5′-CTTACCCTGGTCAGAGGAAGTG-3′	
		Antisense	5′-CCTCAAGGTGAAAGACTGTGCTG-3′	119 bp
*Gapdh*	NM_008084	Sense	5′-CATCACTGCCACCCAGAAGACTG-3′	
		Antisense	5′-ATGCCAGTGAGCTTCCCGTTCAG-3′	153 bp

*Postn*: periostin, *Itgav*: integrin subunit alpha V, *Itgb3*: integrin subunit beta 3, *Itgb5*: integrin subunit beta 5, and *Gapdh*: glyceraldehyde-3-phosphate dehydrogenase.

## Data Availability

The original contributions presented in this study are included in the article. Further inquiries can be directed to the corresponding author. The RNA-seq data generated in this study have been deposited in the NCBI Gene Expression Omnibus (GEO) under accession number GSE313641. Raw sequencing data are available through the NCBI Sequence Read Archive (SRA) and are linked to the GEO record.

## Abbreviations

The following abbreviations are used in this manuscript:

ANOVA	analysis of variance
AR	allergic rhinitis
*ATP5PD*	ATP synthase peripheral stalk subunit D
cDNA	complementary DNA
*COX7A2*	cytochrome c oxidase subunit 7A2
C_T_	threshold cycle
GEO	Gene Expression Omnibus
*GAPDH*	glyceraldehyde-3-phosphate dehydrogenase
hNEC	human nasal epithelial cell
hEGF	human epidermal growth factor
*hsa*	*Homo sapiens*
IgE	immunoglobulin E
IgG	immunoglobulin G
IL-13	interleukin-13
*Itgav*	integrin subunit alpha V
*Itgb3*	integrin subunit beta 3
*Itgb5*	integrin subunit beta 5
KEGG	Kyoto Encyclopedia of Genes and Genomes
*mmu*	*Mus musculus*
mRNA	messenger RNA
*MT-ATP8*	mitochondrially encoded ATP synthase 8
*MT-ND3*	mitochondrially encoded NADH dehydrogenase 3
*MT-ND4L*	mitochondrially encoded NADH 4L dehydrogenase
*MT-ND6*	mitochondrially encoded NADH dehydrogenase 6
*NDUFB3*	NADH:ubiquinone oxidoreductase subunit B3
*NDUFS4*	NADH:ubiquinone oxidoreductase subunit S4
PBS	phosphate-buffered saline
PCR	polymerase chain reaction
*Postn*	periostin
RT	reverse transcription
SRA	Sequence Read Archive
Th2	T helper 2
*UQCRB*	ubiquinol-cytochrome c reductase binding protein
